# Supporting Families of Children With Cancer Using a Virtual Assistant for Social Care: Development and Usability Study

**DOI:** 10.2196/105818

**Published:** 2026-11-13

**Authors:** Emre Sezgin, Adelaide M Booze, Lydia M Wisne, Daniel I Jackson, Micah A Skeens

**Affiliations:** 1 Center for Biobehavioral Health, The Abigail Wexner Research Institute, Nationwide Children's Hospital, 700 Children's Dr, Columbus, OH, 43205, United States, 1 6147223179; 2 Department of Pediatrics, College of Medicine, The Ohio State University, Columbus, OH, United States

**Keywords:** chatbot, conversational AI, pediatric cancer, health-related social needs, caregivers

## Abstract

**Background:**

Families of children with cancer face substantial financial, social, and emotional stressors, including unmet health-related social needs (HRSN) and toxic stress, which contribute to disparities. Virtual assistants (VAs) offer a scalable option to screen for HRSN and connect families to resources, but their design and acceptability in pediatric oncology are not well characterized.

**Objective:**

The objective of this study was to evaluate a VA prototype designed to screen for HRSN and reduce toxic stress among families of children with cancer.

**Methods:**

A 2-phase mixed methods study was conducted to co-design and evaluate a VA prototype for caregivers of children with cancer. In phase 1, a community advisory board (CAB) including health care providers and a parent advocate (n=8) iteratively refined VA content, tone, and interface through 3 virtual co-design sessions. In phase 2, caregivers (n=40) completed in-person VA prototype usability sessions that included structured observation, a semistructured interview, the System Usability Scale (SUS), a caregiver satisfaction survey, and a reaction card task. Social drivers of health, rurality, and neighborhood disadvantage were characterized using survey data and geocoded indices.

**Results:**

CAB feedback refined prototype components including community resources, stress management, conversation history, and resource feedback. In phase 2, usability was high (SUS mean 82.6, SD 14.0); 95% (38/40) of caregivers rated the VA as easy to use and good to use, and 92.5% (37/40) reported that the VA motivated them to remain engaged during screening and resource identification. Qualitative analysis revealed that caregivers favored the VA’s simplicity and its potential to provide timely support, particularly shortly after diagnosis. Participants expressed strong preferences for electronic health record integration to streamline care, though they noted concerns regarding privacy and “app fatigue” as potential adoption barriers. Reaction card responses were predominantly positive (eg, “accessible,” “easy,” and “useful”). Greater socioeconomic vulnerability was associated with higher usability ratings, while rural and medically underserved caregivers reported lower confidence and more negative desirability attributes.

**Conclusions:**

The stakeholder-informed VA to address HRSN and toxic stress in pediatric oncology was highly usable and acceptable in prototype testing. Future work should evaluate live deployment, resource linkage, toxic stress outcomes, and equity in larger, more diverse, and longitudinal studies.

## Introduction

Pediatric cancer remains a leading cause of disease-related death among children; although survival has improved, incidence is rising, and outcome disparities persist [1,2]. There is an emerging need to provide social care support for those with high needs. Health-related social needs (HRSN), including financial, nutritional, and environmental domains, shape a family’s capacity to manage illness and access health care [3]. Chronic exposure to impoverished and unhealthy conditions can produce chronic stress, associated with unmet HRSN and adverse neurodevelopmental and health outcomes across physical, behavioral, socioemotional, and cognitive domains [4,5]. Accordingly, routine screening for unmet HRSN, broader social drivers of health (SDoH), and related mental health needs is critical to identify and address these barriers, reduce disparities, and improve quality of life [6]. Traditional social needs screening is often burdensome and resource-intensive, thus limiting timely, accurate identification and efficient linkage to services, particularly in low-resource clinics [7,8].

In such cases, digital health technologies have shown promise in supplementing health care–related services [9-11]. Within clinics, electronic health record (EHR)–based and tablet PC–facilitated HRSN screenings have been used to support routine screening, and offload social work efforts [9,12]. Virtual assistants (VAs; also known as conversational agents and chatbots) have been emerging for health care communications (eg, mental health screening and social support) [13,14] and community resource access [15,16]. Chatbots provide scalable, personalized, and user-friendly platforms for patient interaction and data collection [17-20].

Built upon our earlier work on a VA designed to screen for unmet HRSN and connect users to community-based resources [16], the objectives of this study were to adapt and evaluate a VA prototype to screen for HRSN and reduce toxic stress among families of children with cancer. Specifically, the study aimed to (1) co-design (refine) the VA prototype with input from key stakeholders and (2) assess its usability and acceptability among caregivers. The VA prototype, in the scope of this study, screens for HRSN, provides personalized referrals (with prepopulated community resources) and offers brief toxic stress support to caregivers of children with cancer, with the aim of providing immediate resource navigation and low-intensity coping strategies.

## Methods

This study consisted of two phases: (1) co-designing the VA prototype with caregivers of children with cancer through a community advisory board (CAB) and using a human-centered iterative design approach, and (2) investigating the VA prototype usability and acceptability through mixed methods user testing.

### VA Prototype

The VA’s conceptual framework was informed by our previous work on an HRSN chatbot developed for pediatric primary care, serving as an exemplar for the VA’s conversational architecture, organization, and subject matter [16]. CAB feedback then guided iterative prototype development, and CAB members reviewed and endorsed the refined prototype for caregiver evaluation in phase 2. Figure 1 outlines the wireframes and iterative refinements across CAB sessions 1 to 3 (Multimedia Appendix 1 details specific design updates). The resulting VA included four main functionalities: (1) a community resource module, (2) a stress management module, (3) a history tab, and (4) a feedback loop. The community resource module began with a brief VA introduction and triaged caregiver needs across food, housing, transportation, and financial resources. After selecting a category, caregivers completed a screening sequence (using prepopulated responses to simulate functionality) that captured ZIP code, dependents’ ages, and child health conditions. The VA then returned a mock resource that caregivers could explore, including options to “save” the resource, and review eligibility criteria, required documentation, benefit options, and application guidance, along with practical details such as address, business hours, cost, phone number, documents, and map links; additional elements (eg, contact information) were available when applicable. The stress management module assessed current affect and stress endorsement. If caregivers indicated stress, the VA administered Functional Impact of Toxic Stress for Parents (FITS-P) [21]–informed questions assessing difficulty managing thoughts, behaviors, schedules, and relationships with children, followed by provision of crisis support information (eg, text line resources) and optional mindfulness exercises. Throughout, caregivers could “learn more,” providing brief definitions and examples for clarification. The mindfulness exercise included guided progressive muscle relaxation and paced breathing supported by visual instructions. The stress management module was designed as a brief, optional, low-intensity supportive feature to acknowledge caregiver stress and provide immediate coping strategies (eg, mindfulness exercises and crisis resources). It was not intended to serve as a comprehensive psychosocial intervention but rather to complement the VA’s primary focus on HRSN screening and resource navigation.

**Figure 1.**
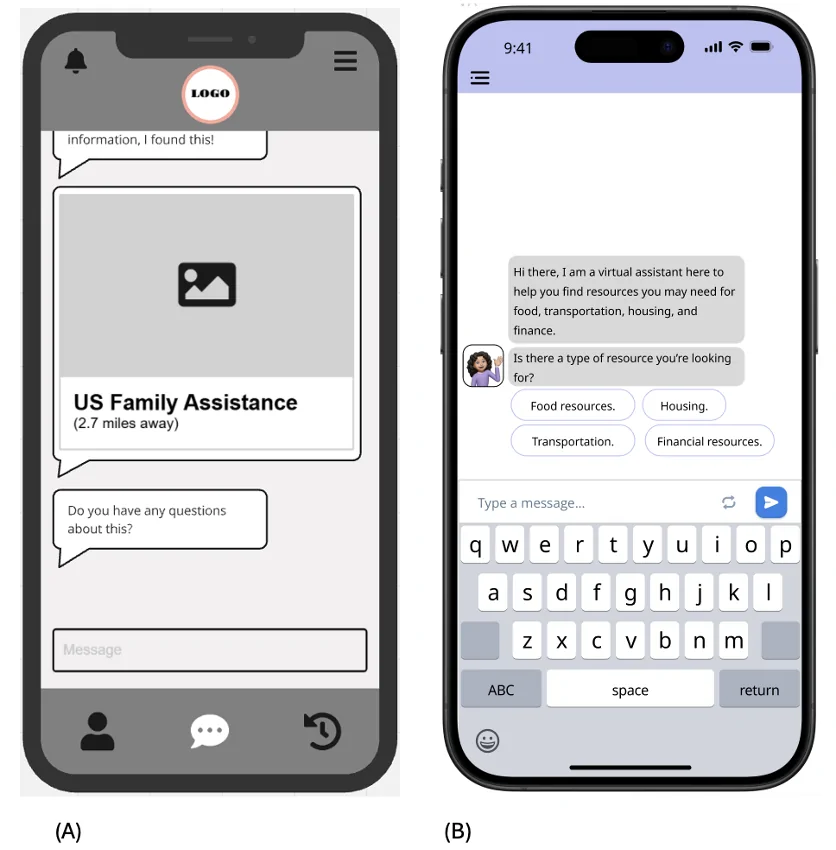
Example (A) wireframe and (B) prototype visuals.

### Phase 1 Study Design

Phase 1 comprised 3 CAB co-design sessions, including participants from a large freestanding children’s hospital and a patient advocate (Figure 2). Each CAB member was selected by the study investigator because of their expertise related to the app’s content and/or their direct engagement with families affected by childhood cancer. The CAB included professionals involved at various stages of the pediatric cancer journey, as well as the head of Momcology, a leading patient advocacy organization supporting caregivers navigating their child’s cancer diagnosis. All CAB members were 18 years or older and provided informed consent prior to participation. Each session was held virtually (40 minutes to 1 hour). During virtual sessions, CAB members reviewed the evolving prototype and offered suggestions. Feedback was iteratively incorporated, and this process continued until consensus was reached for phase 2 testing.

**Figure 2.**
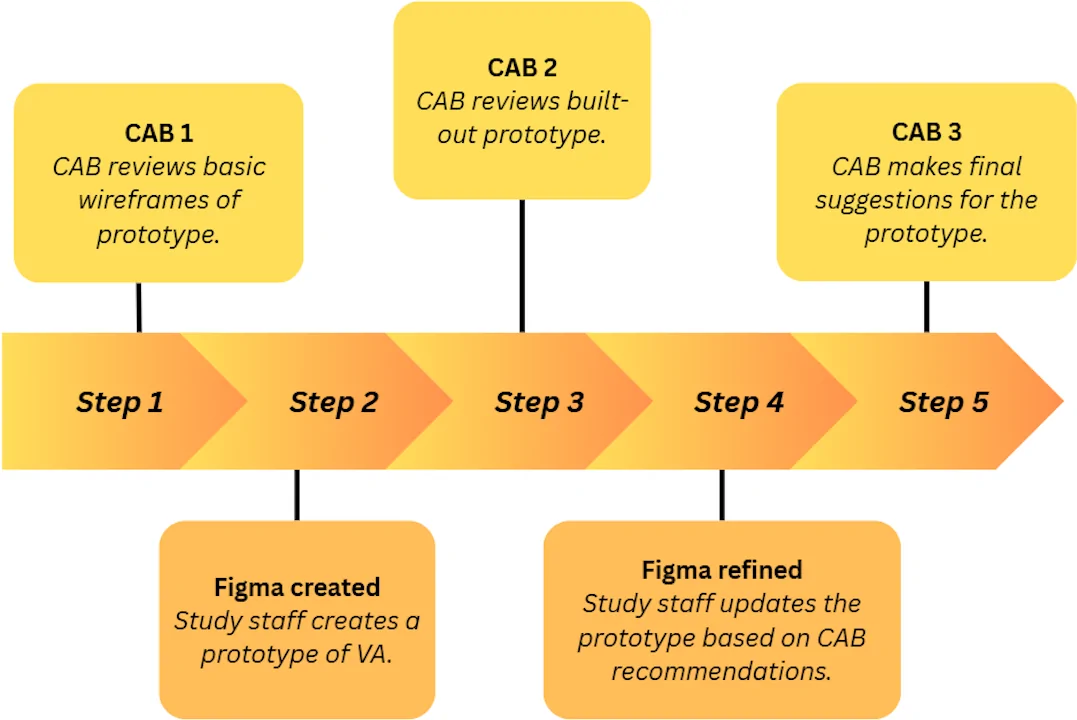
Phase 1 community advisory board (CAB) session progression. “Figma” is an online tool used to create digital prototypes. VA: virtual assistant.

Participants in the initial CAB reviewed VA wireframes via Miro (Bending Spoons S.p.A.), a collaborative design platform. Using an iterative, co-design process, the study team incorporated CAB feedback and developed an interactive prototype using Figma (Figma, Inc), a digital interactive prototyping tool. By the third CAB session, members endorsed the VA’s features and deemed the prototype suitable for presentation to families in phase 2.

The conversational interface of the VA prototype was adapted from the DAPHNE chatbot, a chatbot designed to support families with unmet HRSN and provide personalized, location-based referrals [16]. DAPHNE uses a hybrid conversational architecture and retrieves resources in real time through integration with a social care API [22], which aggregates community-based services such as food, housing, transportation, and financial support. For this study, the chatbot was adapted for pediatric oncology by incorporating context-specific content (prepopulated resources for the ease of evaluation rather than API pull) and a stress management module that evaluated users’ stress level and offered resources based on level of stress.

### Phase 2 Study Design

Phase 2 was a cross-sectional mixed methods evaluation of the VA. Caregivers reviewed the same VA prototype that CAB members refined in phase 1. No changes were made to the prototype between the final CAB session and phase 2, as CAB members approved all items in the prototype. Caregivers were recruited in person from a large Midwestern children’s hospital between October 2024 and February 2025. Eligibility included being aged 18 years or older, being English-speaking, and being a caregiver of a child aged 0 to 17 years with a cancer diagnosis who was receiving active therapy. Of the 57 eligible caregivers approached, 16 declined (most commonly due to limited time, lack of interest, discomfort with research, or a preference for virtual participation), and 41 consented to participate. One caregiver later withdrew, resulting in a final cohort of 40 caregivers. In-person quantitative and qualitative data captured feedback on the VA’s interface, usability, satisfaction, reactions, and content. Caregivers’ engagement and comprehension were assessed using the following steps:

Step 1 involved unobtrusive observation to measure interface intuitiveness (10‐15 minutes). Caregivers were briefly introduced to the VA and asked to start interacting with it. The study team did not intervene or provide instruction but observed and recorded participants’ progress.

Step 2 involved interactive observation (10‐15 minutes). The study team began to interrupt participants during their interaction with the VA by posing questions regarding observed cues that implied success or delay in program use. Caregivers were encouraged to verbalize criticisms of the VA. Since the discussion was based on evolving patterns of interaction, preprescribed questions were not possible.

Step 3 involved debriefing (15‐20 minutes). Caregivers were asked to reflect on their experience with the VA, its interface and contents, and share opinions on strengths and weaknesses. Caregivers completed a 15-minute semistructured interview regarding the VA. They were asked about initial impressions, perceptions of similar tools, feature preferences, perceived helpfulness, missing elements, suggestions, and potential barriers or facilitators of use for their family. Following the interview, caregivers completed the System Usability Scale (SUS), caregiver satisfaction survey, and reaction card.

### Measures

Minimal demographic data were collected during phase 1. Aside from qualitative input, CAB members reported sex, race and ethnicity, age, relationship status, education, employment status, occupation, and work setting. The following measures were collected only from phase 2 participants.

#### Demographic Characteristics

Caregivers reported sociodemographic characteristics consistent with phase 1, along with additional caregiver-child relationship information and child demographics (age, sex, race and ethnicity, and school status and grade). Child diagnosis and treatment were obtained from EHRs.

#### FITS-P

Caregivers completed the 4-item FITS-P [21], reporting dichotomously (yes or no) on each item. Items assessed caregiver difficulty in the past month with (1) managing thinking (eg, intrusive thoughts and poor concentration), (2) regulating behavior (eg, irritability and difficulty with tasks), (3) maintaining schedules (eg, attending work or appointments and managing childcare), and (4) managing the caregiver-child relationship (eg, reduced enjoyment and frustration in caregiving).

#### Phenotypes and Exposures Toolkit

Caregivers completed the phenotypes and exposures measures for SDoH. This instrument provides standardized measurement protocols intended to facilitate the development of effective interventions for mitigating health disparities [23]. The specific measures completed by caregivers included current address, health insurance coverage, food insecurity, health literacy, access to health services, internet access, access to health technology, and perceptions of housing insecurity [24].

#### SUS

Caregivers completed the SUS, a 10-item questionnaire routinely used to assess the functionality and acceptability of digital health applications [25]. Items are rated on a 5-point scale, with scores ranging from 0 to 100. The scale’s reliability (Cronbach α=0.91) and validity (Pearson correlation *r*=0.81 with a 7-point scale of “user-friendliness”) are well-established [26]. A score exceeding 68 is considered above average [27].

#### Caregiver Satisfaction

Caregiver satisfaction was evaluated using a 9-item survey developed by the investigators to ascertain caregiver perspectives on the VA. This included perceived benefits, burdens, barriers, suggested modifications, and overall satisfaction. Items were scored on a 4-point scale, with higher total scores indicating greater satisfaction [28].

#### Reaction Card

VA desirability was assessed using a reaction card method from Microsoft’s “desirability toolkit” [29]. Caregivers selected from 55 predefined descriptors and selection frequencies were summarized to characterize overall user impressions. A higher incidence of positive words vs negative words indicates higher vs lower usability [30].

#### Rural Residency

Caregiver addresses were categorized as rural or nonrural using Rural-Urban Commuting Area (RUCA) codes from the 2020 census. Rural residence was defined as RUCA 4 to 10 (micropolitan to rural), and nonrural as RUCA 1 to 3 (metropolitan) consistent with prior research [31,32].

#### SDoH

Participant addresses were coded for SDoH to inform whether contextual, nonmedical factors related to residence were associated with VA usability. The Social Vulnerability Index (SVI) uses 16 variables across 4 domains (socioeconomic status, household characteristics, racial and ethnic minority status, and housing and transportation) [33]. SVI scores range continuously from 0 to 1, with higher scores indicating greater vulnerability. Addresses were also coded for medically underserved area (MUA) designations (0=served, 1=underserved) [34] and Appalachian county status using Appalachian Regional Commission county lists (0=non-Appalachian, 1=Appalachian) [35].

### Statistical Analysis

Data were analyzed using SPSS (version 28; IBM Corp) for Windows. For the CAB, descriptive statistics (frequencies, means, percentages, and SDs) summarized demographic data. Semistructured interviews were transcribed verbatim, and rapid analyses were conducted to extract major themes [36]. For phase 2, descriptive statistics, bivariate correlations, and 2-tailed *t* tests summarized quantitative data. Sample sizes for the analyses varied across correlations because geocoded indices could be derived for 37 of 40 addresses. Furthermore, individual survey items had occasional missing responses, and the contributing sample size was reported alongside each coefficient.

Semistructured interviews were transcribed verbatim for content analysis and coded using NVivo software (Lumivero). We conducted a directed content analysis, using an initial codebook developed from prior literature on digital health usability and acceptability [37,38]. This deductive framework guided early coding while allowing for the identification of novel concepts emerging from the data.

Two independent coders applied the preliminary codebook to an initial subset of transcripts (n=10) to assess code applicability and completeness. During this process, the coders identified content not adequately captured by the a priori codes. Through discussion and consensus, the codebook was iteratively refined to incorporate additional inductive codes (eg, “timing of intervention” and “preferred modality”) to better reflect participants’ perspectives—particularly in response to questions about when and how caregivers would find the intervention most useful.

Following codebook refinement, all transcripts were coded independently by the 2 coders. Discrepancies were resolved through discussion until consensus was reached, and the finalized codebook was applied consistently across the dataset.

After coding was complete, the coders collaboratively reviewed coded data to identify patterns and generate themes. These preliminary themes were then discussed with the broader study team to ensure consistency with the data and to enhance analytic rigor. Final themes were agreed upon through team-based consensus.

### Ethical Considerations

The study was approved by the Nationwide Children’s Hospital Institutional Review Board (STUDY00003766) and was designed in accordance with the ethical standards laid out by the Declaration of Helsinki. Eligible interested participants provided informed consent prior to enrollment and were assigned ID numbers for confidentiality. All participant information was anonymized in this paper, including the text, tables, and figures. Upon completion of the study, all participants (caregivers) received a US $25 gift card as compensation. CAB members received US $250 for their time.

## Results

### Phase 1 Results

Eight individuals participated in the phase 1 CAB. There were 2 social workers; and 1 of each of the following: nurse, pediatric psychologist, physician, administrator, and psychosocial services director; and 1 Momcology community advocate. See Multimedia Appendix 1 for CAB demographics.

### Phase 1 Qualitative Data

Four major themes emerged from CAB sessions: (1) personalization and integration with the medical team to facilitate engagement, (2) language and technology accessibility as potential barriers to engagement, (3) simple and empathetic language to frame the intervention, and (4) easy-to-navigate presentation. Using an iterative design approach, changes were made to the prototype based on CAB member suggestions. CAB exemplar quotes are shown in Table 1, and feedback implementation examples are shown in the Multimedia Appendix 1. In the final CAB, members endorsed the VA, which indicated the prototype was ready for phase 2.

**Table 1.** Exemplar quotes from community advisory board (CAB) sessions.

Suggestion	Exemplar quotations
Framing content and language	
CAB member	“I think the prompting’s going to be key” “You guys may want to consider phrasing it in a sort of a conversational directed at the person responding...trying to personalize it a little bit.” “We could say ‘it’s good to hear that you’re managing your stress well at this time.’ Something like that.”
Perceived facilitators to engagement	
Personalization	“Will it know your name?...It makes it feel personable as opposed to, you know, a bot.”
Integration with medical team	“I think it being integrated is a great idea, because then it’s like one less thing for the families to have to remember to tell their provider.”
App analytics, reminders, and history	“The next time they go in, she could say ‘during our last chat, during our last conversation, I offered this resource. Did you have an opportunity to use it? Was it helpful to you?’”
Potential barriers to engagement	“I’m also wondering availability in other languages and like what reading level it’s being written at.” “One of the most mentioned things among our very stressed population that they cannot get to a mental health provider.” ”And I think being very thoughtful about how often resources are updated, so I think we could potentially lose trust quickly if families are referred spaces that either aren’t available or, um, you know.” “If families aren’t using her because there is a report back or they’re afraid that there that you know they want to use the resource...they’re not asking their social worker intentionally, right? Umm, are we actually creating a barrier accidentally?”
Suggestions on presentation and visuals	“I think we should always be ending with ‘do you need help with other resources?’ And then it takes them back to those prompts. They might not understand to click that refresh button and then start the conversation over.” “looking at those three buttons, I think as a as a layman user, I would have a hard time identifying if I needed to, which one I needed to do.”

### Phase 2 Quantitative Results

In phase 2, 41 caregivers consented to participate; 1 withdrew following consent, resulting in a final sample of 40. Caregivers were mostly female (n=33, 82.5%); White (n=34, 85%); non-Hispanic, Latino, or Spanish (n=36, 90%); married (n=27, 67.5%); Christian (n=29, 72.5%); and had at least a high school–level education (n=13, 32.5%). Caregivers were split between employed working full time (n=13, 32.5%) and not employed and not looking for work (n=13, 32.5%), and most made more than US $50,001 a year (n=27, 67.5%). Children were mostly male (n=25, 62.5%); White (n=36, 90%); not Hispanic, Latino, or of Spanish origin (n=34, 85%); and were homeschooled (n=12, 30%). Detailed demographics are reported in Table 2.

**Table 2.** Demographic and clinical characteristics of caregivers and children in phase 2 (N=40).

Baseline characteristic	Participants
Age (y), mean (SD)	39.6 (11.35)
Sex, n (%)	
Female	33 (82.5)
Male	7 (17.5)
Race, n (%)	
Black or African American	2 (5)
Filipino	1 (2.5)
Mixed race	1 (2.5)
Some other race	2 (5)
White	34 (85)
Hispanic ethnicity, n (%)	
No, not of Hispanic, Latino, or Spanish	36 (90)
Yes, Mexican, Mexican American, or Chicano	3 (7.5)
Yes, Puerto Rican	1 (2.5)
Marital status, n (%)	
Single	7 (17.5)
Married	27 (67.5)
Divorced	2 (5)
Separated	2 (5)
Living with someone	2 (5)
Occupation, n (%)	
Employed, working full time	13 (32.5)
Employed, working part time	5 (12.5)
Employed, working more than one job	2 (5)
Not employed, looking for work	2 (5)
Not employed, not looking for work	13 (32.5)
Retired	1 (2.5)
Not listed	4 (10)
Work situation, n (%)	
Work in location outside of home	16 (40)
Split work from home and outside of home	4 (10)
Primarily work from home	5 (12.5)
Not employed or not applicable	15 (37.5)
Education, highest grade completed, n (%)	
High school	13 (32.5)
Post high school (technical or trade school)	3 (7.5)
College	12 (30)
Graduate or professional	12 (30)
Household income (US $ per y), n (%)	
Less than 25,000 per year	3 (7.5)
25,001-50,000	10 (25)
50,001-75,000	6 (15)
75,001-100,000	7 (17.5)
100,001-150,000	8 (20)
150,001 or more	6 (15)
Child’s diagnosis	
Leukemia	19 (47.5)
Lymphoma	5 (12.5)
Solid tumor	13 (32.5)
Central nervous system	2 (5)
Missing	1 (2.5)
Social Vulnerability Index, mean (SD)	0.34 (0.29)
Rural-Urban Commuting Area score, mean (SD)	2.68 (2.58)
Medically underserved area, n (%)	13 (32.5)
Appalachian residency, n (%)	5 (12.5)

### Usability

The mean SUS score was 82.62 (SD 14.01), with a median of 82.50 (IQR 72.50-97.50). Most caregivers reported that the VA was useful (38/40, 95%), and that others could learn to use it quickly (37/39, 94.9%, 1 caregiver did not respond to this item; Figure 3). Bivariate correlations indicated lower socioeconomic status (SVI subcomponent) was associated with higher usability ratings (*r*=0.368, n=37; *P*=.02). Overall SVI showed a similar trend; greater vulnerability was associated with higher ratings, although the association was not statistically significant (*r*=0.317, n=37; *P*=.06). Correlations with individual items from the SUS revealed caregivers from a rural area were more likely to report they need the support of a technical person to use the VA (*r*=–0.382, n=40; *P*=.02) and felt less confident using the VA (*r*=–0.419, n=39; *P*=.008). Independent 2-tailed *t* tests comparing binary SDoH variables and overall SUS scores showed Appalachian residents were somewhat more likely to rate the VA as more usable compared with non-Appalachian residents (Appalachian: mean 89.50, SD 13.39; non-Appalachian: mean 81.64, SD 14.01), although this difference was not only moderately significant (*t*_38_=1.64; *P*=.05).

**Figure 3.**
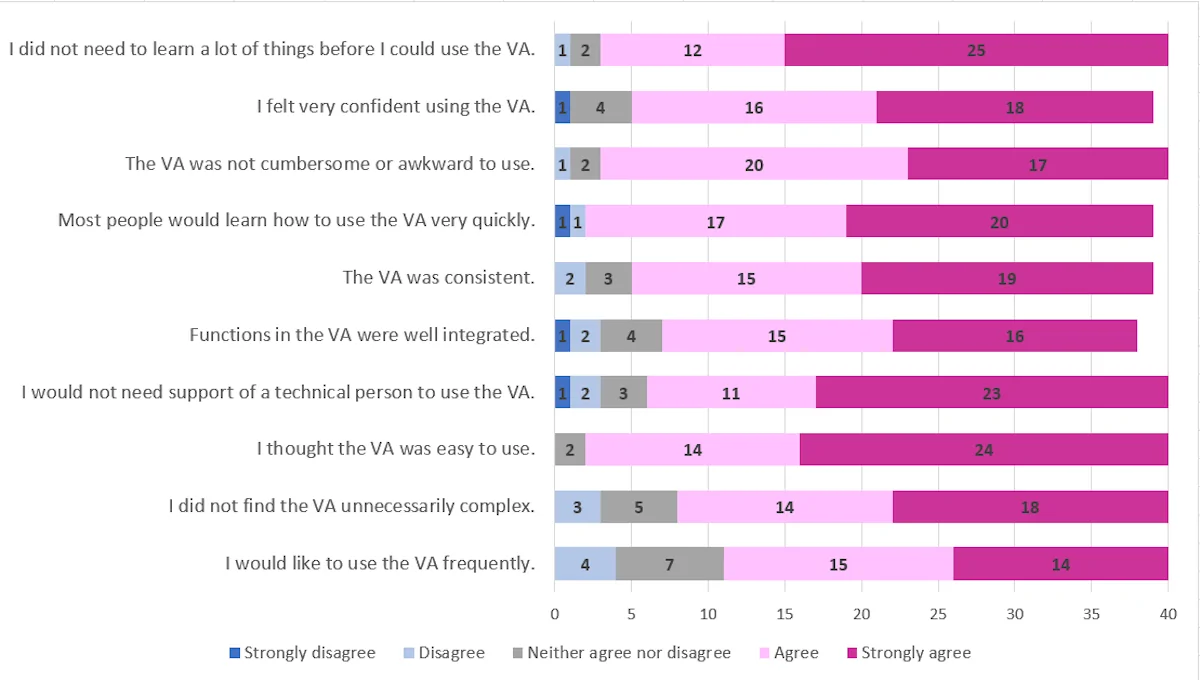
Virtual assistant (VA) System Usability Scale (SUS) survey responses from caregivers.

### Caregiver Satisfaction

Caregivers frequently rated the VA as easy to use (38/40, 95%) and good to use (38/40, 95%). Most also reported that the VA motivated them to remain engaged during screening and in finding community resources (37/40, 92.5%; Figure 4). Bivariate correlations and independent 2-tailed *t* tests between individual items on the caregiver satisfaction survey and SDoH variables were nonsignificant.

**Figure 4.**
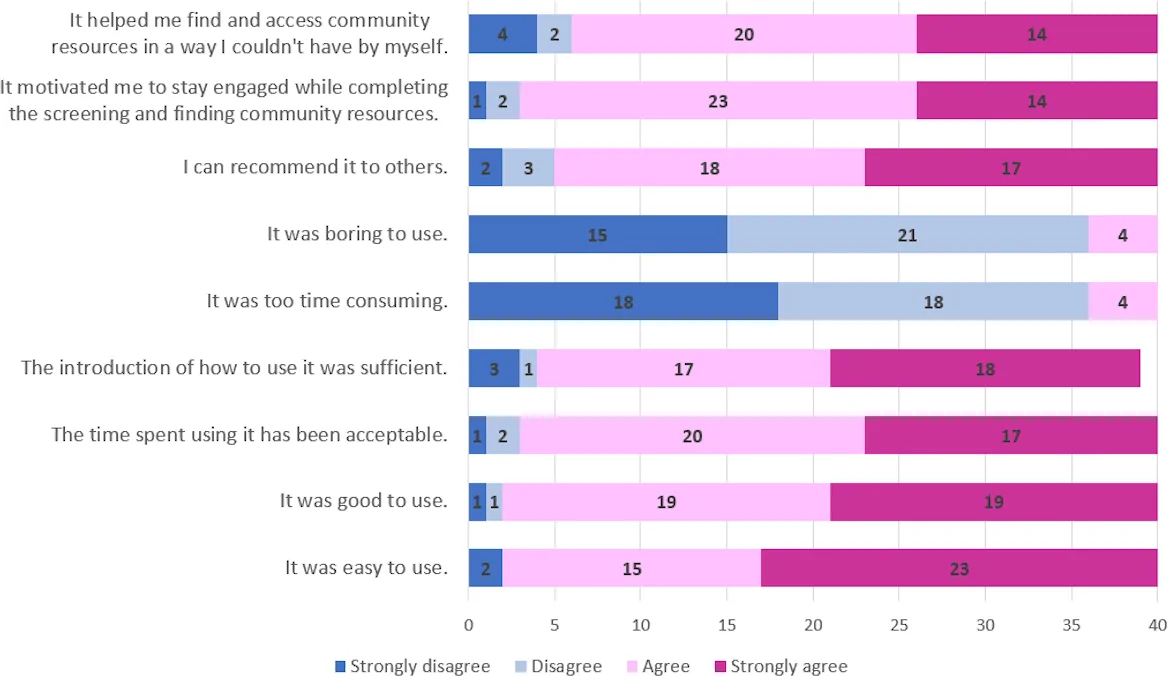
Caregiver satisfaction survey responses.

### Reaction Cards

Most caregivers reported positive experiences with the VA; accessibility (34/40, 85%), ease of use (34/40, 85%), and usefulness (32/40, 80%) represented the highest-rated reactions (Figure 5). Bivariate correlations between reaction card items and SDoH were conducted. Caregivers from rural areas rated the VA as less collaborative (*r*=-0.403; n=40; *P*=.01) and less straightforward (*r*=–0.320; n=40; *P*=.04) compared with nonrural caregivers. Those residing in MUAs rated the VA as less attractive (*r*=–0.489; n=40; *P*=.001), less empowering (*r*=–0.349; n=40; *P*=.03), more intimidating (*r*=0.331, n=40, *P*=.04), and less useful (*r*=–0.320; n=40; *P*=.04) than caregivers from medically served regions.

**Figure 5.**
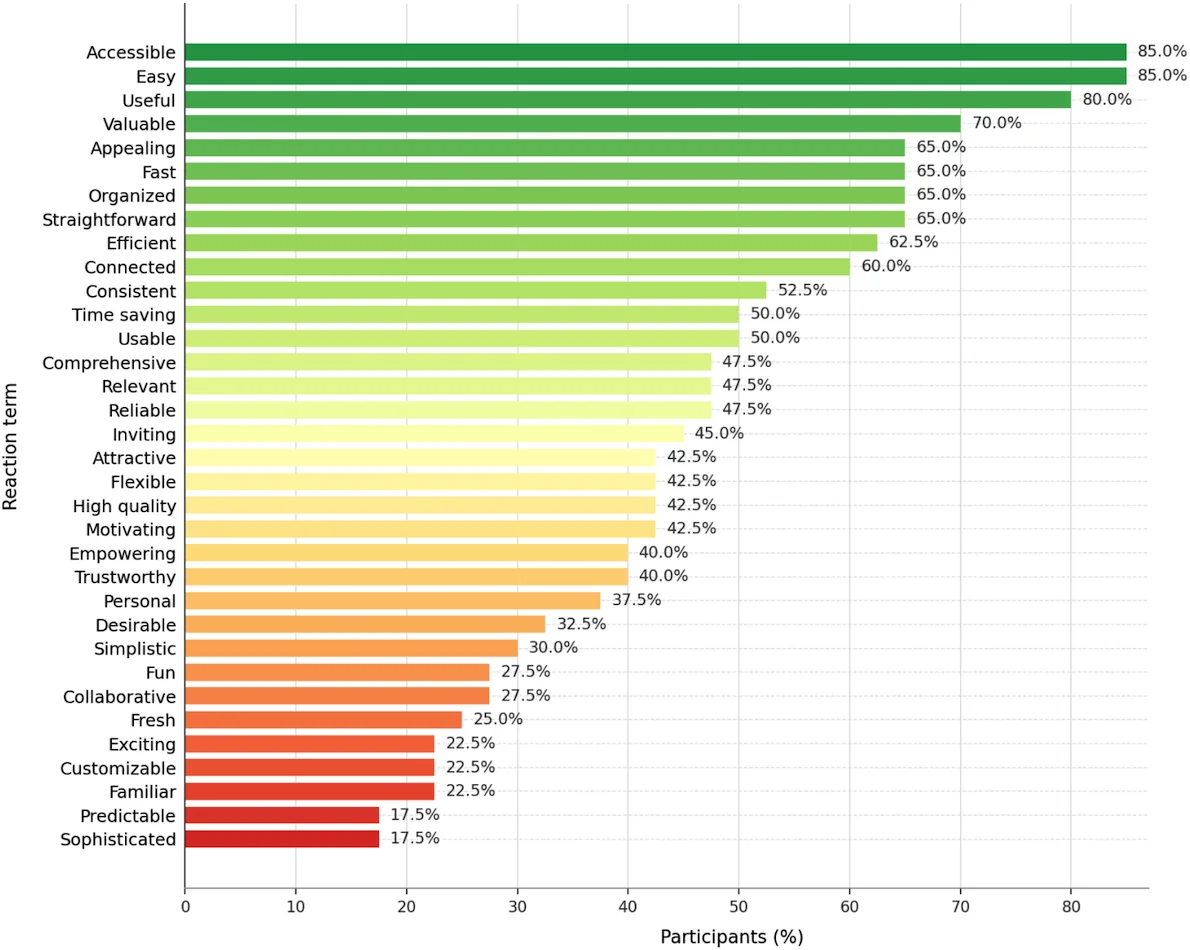
Reaction card results. Reaction terms selected by fewer than 10% are excluded from the figure: confusing (7.5%), busy (5%), complex (5%), intimidating (5%), not valuable (5%), gets in the way (2.5%), inconsistent (2.5%), overbearing (2.5%), rigid (2.5%), stimulating (2.5%), time consuming (2.5%).

### Phase 2 Qualitative Results

Qualitative analyses resulted in three primary themes: (1) the acceptability of the VA, attributed to its simple and effective design; (2) caregiver preferences regarding optimal VA usefulness; and (3) the applicability of VA resources to caregivers’ real-time needs.

#### VA Acceptability Through Simple, Effective Design

Caregivers consistently reported that the VA prototype’s quality (ie, personalization, clarity, presentation, attractiveness, functionality, and completeness) contributed to ease of use and utility in addressing current needs. The VA’s clear prompts, straightforward navigation, and appealing layout fostered trust and usability. For instance, one caregiver explained, “the prompts were helpful. It makes it quick,” while another remarked, “the pictures are helpful by showing the movements,” when referring to the stress management module. Ease of navigation also enhanced engagement, as one caregiver noted, “it was easy to navigate through. And um, pretty self-explanatory, you know what I mean? Which is good,” while another commented, “I think the straightforwardness of it is really nice and helpful.” Caregivers further emphasized the value of personalization, with one individual suggesting, “people have different aesthetics,” which indicates potential for customizable features.

#### Caregiver Preferences for Optimal VA Usefulness

Caregivers frequently offered suggestions regarding intervention timing, format, module preferences, nontechnology preferences, and general recommendations. Many endorsed the VA would be most beneficial shortly after diagnosis. For example, one caregiver remarked, “it would have been very useful, especially at the very beginning of when he got- first got diagnosed.” While the app format was generally favored by caregivers, as reflected in one caregiver's comment, “I think it’s just way easier to just have the app on your phone,” some caregivers expressed disinterest in using an app, noting, “I don’t want to download another app.” Caregivers also advocated for integration with existing health systems, as exemplified by comments such as, “if it knew my schedule...if it’s connected to MyChart,” and for broader content coverage, requesting, “list some more resources or something. For the whole family, not just me.”

#### Applicability of VA Resources to Caregivers’ Real-Time Needs

Caregivers emphasized the accessibility, applicability, and relevancy of the VA, as well as whether they already possessed a system for addressing social needs. Related to accessibility, one caregiver stated, “I don’t think that I would have any barriers. Um, all you really need is like a phone, internet connection um which we have, um a lot of places even if you don’t have Wi-Fi you can access it on.” Perceptions of access were not uniform, however, and caregivers in rural and MUAs more often described needing technical assistance to use the VA confidently. Caregivers also highlighted the VA’s potential to offer timely assistance during a cancer diagnosis, with one individual stating, “it would be very helpful, for, um, patients and their families, especially their parents that are going through big transitions. Like, I went through.” Furthermore, caregivers reflected on unmet needs and the VA’s capacity to fulfill them: “we needed financial resources, I mean, so, had this been readily available to us, it would have been easier for us to access the resources that we needed.” Moreover, caregivers underscored how the VA conserved time and effort compared with general searches. “I liked it because I am a googler...but this is, a little more specific with the needs of someone in our situation, what we’re dealing with, so I can just easily go to the app instead of having the whole world of google to navigate through.”

## Discussion

### Principal Findings

This 2-phase mixed methods study adapted and evaluated a stakeholder-informed VA prototype to support social care in pediatric oncology by screening for unmet HRSN and offering brief toxic stress support for caregivers. Phase 2 findings reflected caregiver interaction with a semifunctional prototype that included mock-up resource outputs. Within that context, the co-design process was a notable strength. Input from a multidisciplinary CAB and community advocate refined tone, navigation, resource presentation, history functions, and pathways back to the care team before caregiver testing. These prototype refinements likely contributed to the high usability (SUS score=82.62) and acceptability observed in phase 2 and are consistent with prior work, emphasizing the importance of co-design in creating digital health tools that are engaging and meet the specific needs of end users [39-44] and caregivers of children with cancer [44-48].

At the same time, subgroup patterns highlighted important equity considerations for future implementation. Rural caregivers more frequently reported needing technical support and lower confidence: “it would be nice to have that customer service...where they could help you over the phone...or a tutorial.” Additionally, caregivers residing in MUAs perceived the VA prototype as less attractive, less empowering, and more intimidating: “you’re talking to a database...I’m just not comfortable talking to [something] like that.” These findings suggest that a well-performing VA may still require tailored onboarding, content suitable for users with low-literacy levels and multilingual content, multimodal access, and optional human assistance to ensure equitable adoption across geographic and resource contexts [45,47,48].

### Interpretation and Implications for Social Care

Our findings should be interpreted within a rapidly evolving pediatric oncology HRSN landscape. Increasingly, institutions are implementing social needs and financial hardship screening through social work assessment, direct caregiver questionnaires, patient-reported outcomes, and patient portal or EHR [13]. Recent pediatric oncology data suggest systematic screening is feasible using multipronged workflows, and NCI Community Oncology Research Program data indicate that many pediatric oncology practices already routinely screen for financial distress and respond through social work or navigation services [49,50]. In this context, a VA would ideally complement rather than replace existing institutional processes and providers [51]. A VA prototype may be especially useful for extending support between visits, organizing information at high-burden times such as diagnosis, standardizing first-line education, collecting structured follow-up on whether shared resources were used, and triaging unresolved or complex concerns back to social work, financial navigation, or the oncology team [52].

How the VA would maintain accurate resource lists when community programs have fluctuating availability and time-varying eligibility criteria is a major implementation uncertainty, similar to other digital health technologies [12]. In the present study, phase 2 caregivers responded to curated resource lists and prepopulated details. In our earlier DAPHNE architecture [15,16], live deployment was envisioned through API connections to established community resource platforms rather than a static, manually curated list. However, API connectivity alone will not eliminate the underlying volatility of community resources. We acknowledge that a production version would still require routine synchronization with source databases, local stewardship of high-priority pediatric oncology resources, and a human-in-the-loop feedback process so caregivers and staff can flag closed programs, inaccurate hours, or changing eligibility. We view the VA as a decision support and referral aid that should surface curated institutional, community, and public-benefit resources relevant to a family’s location and screening responses, but not as an autonomous system for making final determinations about nuanced HRSN [53]. Institution-specific supports (eg, hospital food banks, parking or lodging assistance, and philanthropy funds) and government-mandated services (eg, Medicaid transportation supports) would likely need local configuration and escalation pathways to social work for confirmation [54,55].

Integration with the EHR also warrants dedicated consideration, given that participants in both phases identified it as both a potential facilitator of use and an area of concern. Participants viewed EHR integration as a way to reduce repeated disclosure, improve continuity with the care team, and embed social care support within familiar tools such as patient portals. However, they also raised concerns about privacy, stigma, surveillance, and the possibility that families might assume that the VA was continuously monitored by clinicians or intended for medical questions. Future EHR-linked deployment should therefore use explicit consent, clearly communicate what information is shared and with whom, distinguish social care triage from clinical messaging, and preserve an option for confidential self-directed use when families prefer not to disclose every interaction [16,56,57].

### Limitations and Future Directions

A notable limitation is that the prototype used in phase 2 included prepopulated, mock-up resources rather than live, generated content. As a result, caregivers interacted with a simulated environment that may not capture the complexity and technical constraints of real-world use. Consequently, our findings regarding usability and acceptability may overestimate how the VA would perform under real-world conditions. Additional limitations include a relatively small and demographically homogenous CAB and caregiver sample, single-site recruitment, cross-sectional evaluation, and lack of a comparator, which limited generalizability and precluded conclusions about clinical effectiveness. Although the CAB included a parent advocate who had navigated their child’s cancer diagnosis, its composition was determined by professional expertise rather than by correspondence with the caregiver sample, and the co-design process may therefore have underweighted the daily financial and logistical constraints facing lower-income families. Therefore, subsequent iterations should recruit caregivers with lived experience of unmet social needs as co-designers alongside clinical experts. In addition, future studies should evaluate the VA longitudinally in more diverse populations, test clinical effectiveness and cost-effectiveness in randomized trials, and incorporate caregiver-prioritized enhancements such as personalization and EHR integration. While the VA included a stress management component, it is important to note that this feature was not designed to replace evidence-based, longitudinal psychosocial interventions commonly used in pediatric oncology care. Rather, it served as a brief, embedded support to acknowledge caregiver distress and offer immediate guidance within the context of social needs screening. Future work should evaluate how such low-intensity digital supports may complement, rather than substitute for, comprehensive psychosocial care models. Addressing the digital divide identified in this study should be a priority for subsequent implementation, including strategies to support device and internet access for families in need, as well as offering tailored training and technical support. Future work should also evaluate the VA’s applicability in other pediatric chronic illness populations facing similar social and psychosocial needs.

### Conclusions

This study provides initial evidence for the acceptability and usability of a stakeholder co-designed VA prototype to support social care for caregivers of children with cancer. Findings suggest particular promise for reaching families with greater social vulnerability, while underscoring the need to address barriers to equitable digital access and support to ensure equitable implementation. With further real-world evaluation and refinement, this approach may become scalable across pediatric oncology and care for children with other chronic illnesses.

## Supplementary material

10.2196/105818 Multimedia Appendix 1 Phase 1 demographics and iterative design updates.

## Abbreviations

CAB: community advisory board
EHR: electronic health record
FITS-P: Functional Impact of Toxic Stress for Parents
HRSN: health-related social needs
MUA: medically underserved area
RUCA: Rural-Urban Commuting Area
SDoH: social drivers of health
SUS: System Usability Scale
SVI: Social Vulnerability Index
VA: virtual assistant
