# Supplementary material for: Supporting Families of Children With Cancer Using a Virtual Assistant for Social Care: Development and Usability Study
Source: JMIR Cancer. 2026 Nov 13;12:e105818. doi: 10.2196/105818 (PMC13761665; doi:10.2196/105818)
Supplement: Supplementary file 1 [file cancer-v12-e105818-s001.pdf]

# Supplementary Materials

## SM1. Phase 1: demographic characteristics of CAB members

| Baseline Characteristics | | N (%) or M (*SD*) |
|---|---|---|
| Participant Age | | 47.1 (*5.4*) |
| Participant Gender | | |
| | Female | 8 (100%) |
| Participant Race | | |
| | White | 8 (100%) |
| Participant Hispanic Ethnicity | | |
| | Not Hispanic | 8 (100%) |
| Participant Marital status | | |
| | Married | 7 (87.5%) |
| | Divorced | 1 (12.5%) |
| Participant Occupation | | |
| | Employed, working full-time | 7 (87.5%) |
| | Employed, working part-time | 1 (12.5%) |
| Participant Work Situation | | |
| | Work in location outside of home | 6 (75%) |
| | Primarily work from home | 1 (12.5%) |
| | Not employed/Not applicable | 1 (12.5%) |
| Participant Education, Highest Grade Completed | | |
| | College | 2 (25%) |

| | | |
|---|---|---|
| | Graduate/Professional | 6 (75%) |
| Household Income | | |
| | $100,001 - $150,000 per year | 3 (37.5%) |
| | $150,001 or more | 5 (62.5%) |

**SM2. CAB feedback and app implementation**

| Feedback from CAB | Implementation | |
|---|---|---|
| Including a crisis line in managing stress module (CAB 3) | A link to a crisis line was added when users indicated high stress | 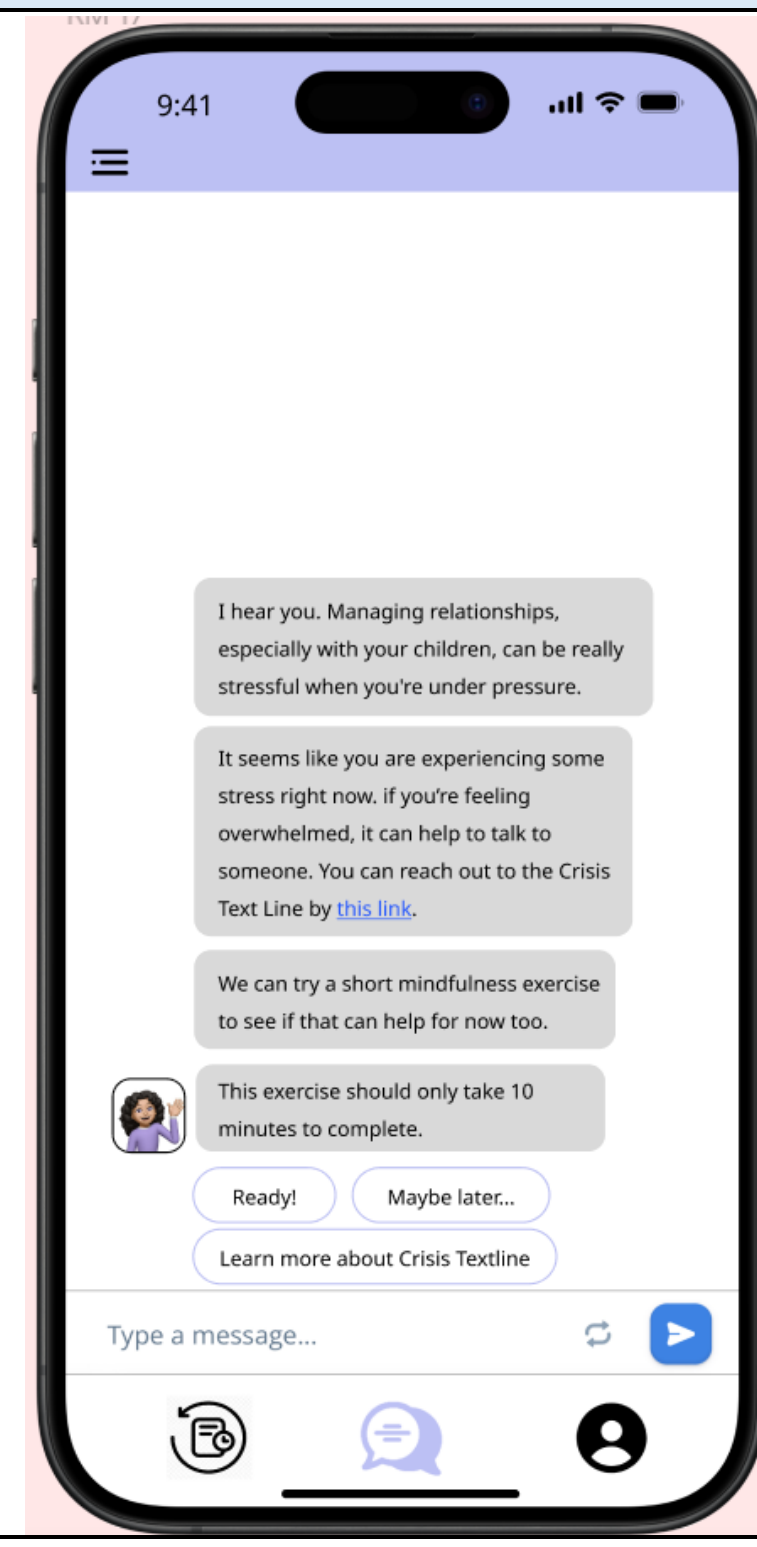 |

| | | |
| --- | --- | --- |
| Opportunity for users to provide feedback on helpfulness of resources | Study team incorporated a prompt for feedback after every conversation | 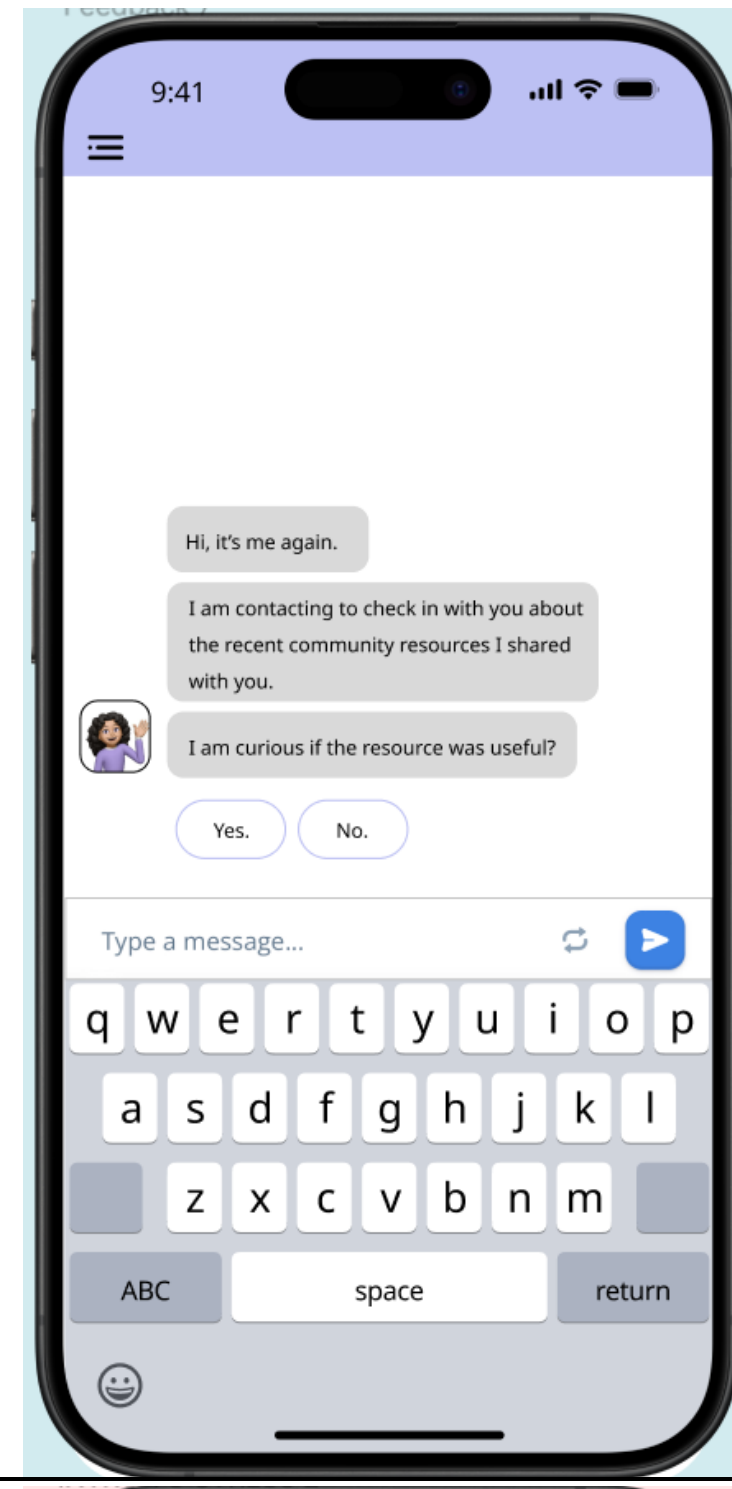 |
| VA should offer stress management tool even when screening results in "low stress" | Mindfulness and breathing exercises were added that appeared regardless of level of stress | 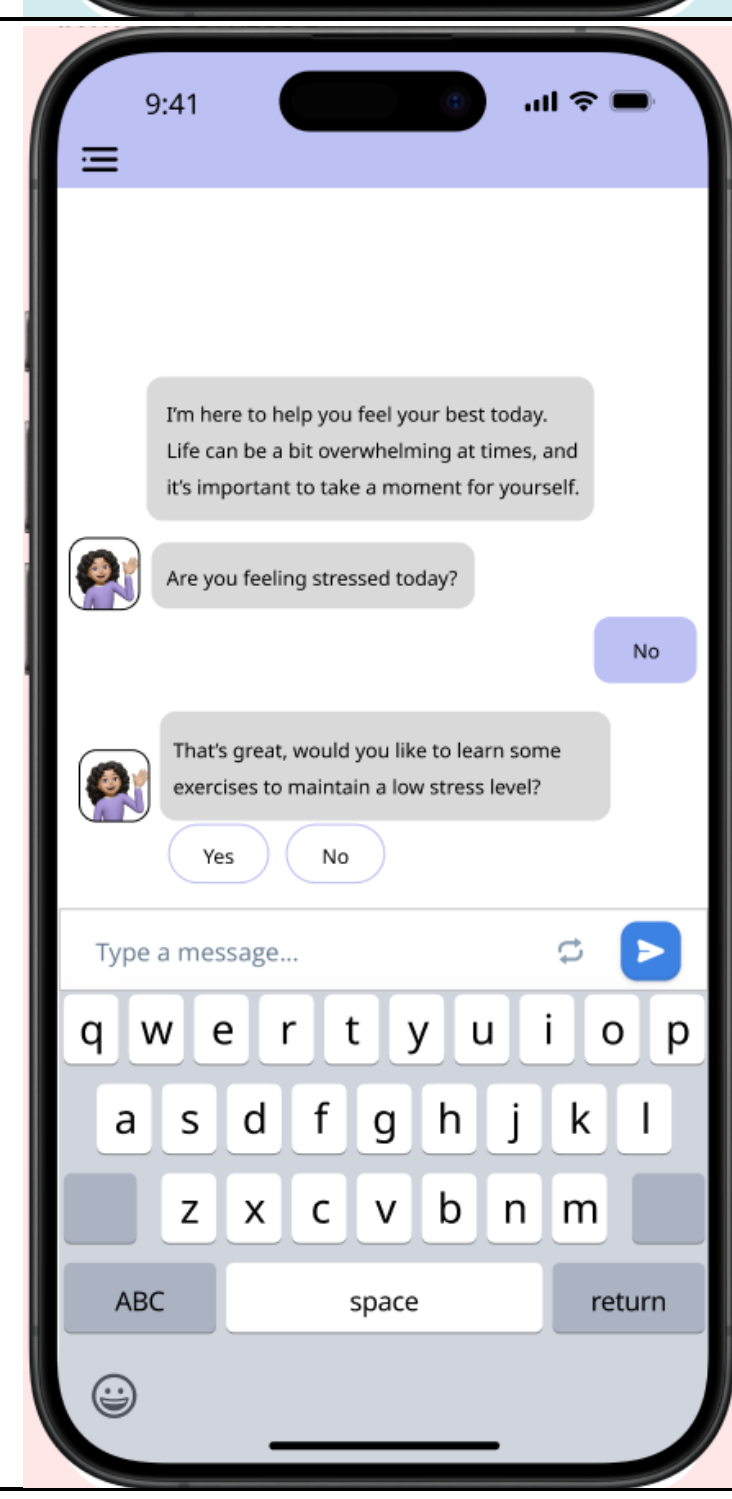 |

<table>
<tr>
<td>Creating an intuitive way to decide what to do next when finding resources</td>
<td>Study team added prompts when opening the finding resources module to suggest possible resources for users</td>
<td>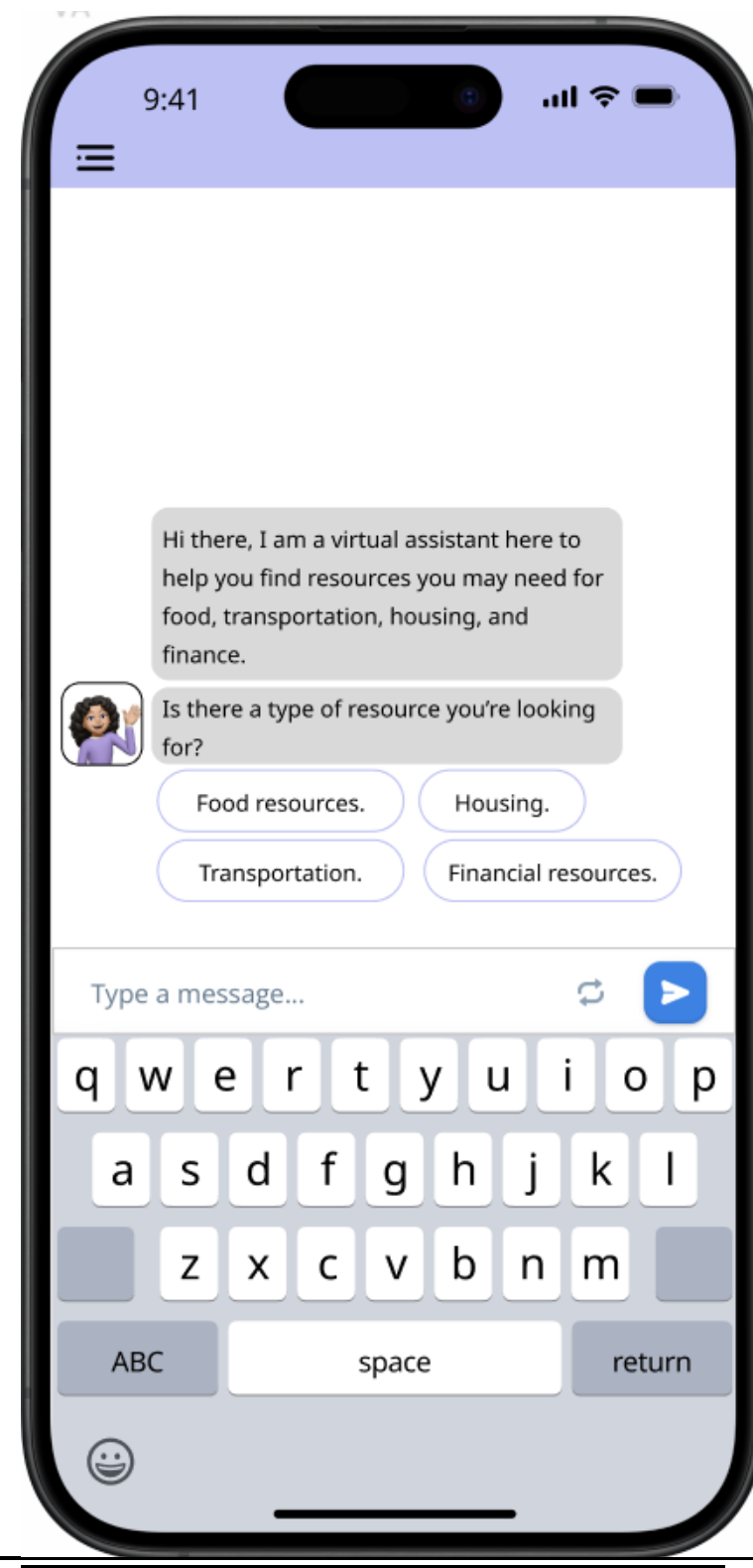
</td>
</tr>
<tr>
<td>Keeping a report of what the user has searched before so they can reference back</td>
<td>A history tab was added to the prototype home page, as well a favorite-ing feature</td>
<td>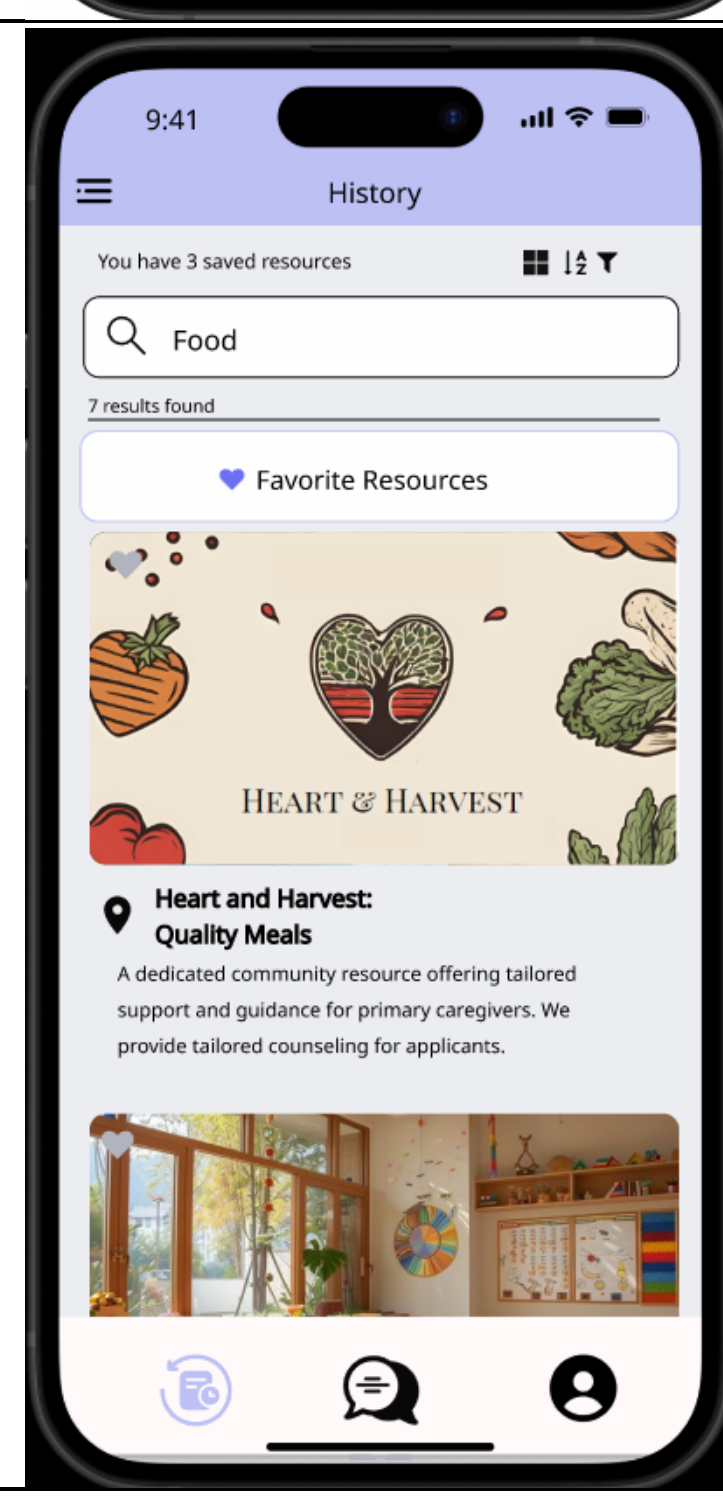
</td>
</tr>
</table>
